# Progress in the Metabolic Engineering of *Yarrowia lipolytica* for the Synthesis of Terpenes

**DOI:** 10.34133/bdr.0051

**Published:** 2024-11-12

**Authors:** Shun-Cheng Liu, Longxing Xu, Yuejia Sun, Lijie Yuan, Hong Xu, Xiaoming Song, Liangdan Sun

**Affiliations:** ^1^ Hebei Key Laboratory for Chronic Diseases, Tangshan Key Laboratory for Preclinical and Basic Research on Chronic Diseases, School of Basic Medical Sciences, North China University of Science and Technology, Tangshan 063210, Hebei, China.; ^2^ Health Science Center, North China University of Science and Technology, Tangshan 063210, Hebei, China.; ^3^ Key Laboratory for Quality of Salt Alkali Resistant TCM of Hebei Administration of TCM, North China University of Science and Technology, Tangshan 063210, Hebei, China.; ^4^ Inflammation and Immune Diseases Laboratory of North China University of Science and Technology, Tangshan 063210, Hebei, China.; ^5^ School of Nursing and Rehabilitation, North China University of Science and Technology, Tangshan 063210, Hebei, China.; ^6^ School of Life Sciences, North China University of Science and Technology, Tangshan 063210, Hebei, China.; ^7^ North China University of Science and Technology Affiliated Hospital, Tangshan 063000, Hebei, China.; ^8^ School of Public Health, North China University of Science and Technology, Tangshan 063210, Hebei, China.

## Abstract

Terpenes are natural secondary metabolites with isoprene as the basic structural unit; they are widely found in nature and have potential applications as advanced fuels, pharmaceutical ingredients, and agricultural chemicals. However, traditional methods are inefficient for obtaining terpenes because of complex processes, low yields, and environmental unfriendliness. The unconventional oleaginous yeast *Yarrowia lipolytica*, with a clear genetic background and complete gene editing tools, has attracted increasing attention for terpenoid synthesis. Here, we review the synthetic biology tools for *Y. lipolytica*, including promoters, terminators, selection markers, and autonomously replicating sequences. The progress and emerging trends in the metabolic engineering of *Y. lipolytica* for terpenoid synthesis are further summarized. Finally, potential future research directions are envisioned.

Terpenes, also known as isoprenoids, are a class of compounds containing isoprene (C_5_H_8_) as the basic structural unit; they can be categorized into hemiterpenes (C_5_), monoterpenes (C_10_), sesquiterpenes (C_15_), diterpenes (C_20_), triterpenes (C_30_), and tetraterpenes (C_40_) [1]. Terpenes are important plant substances that are involved in signal transduction, environmental adaptation, defense, and growth [2,3]. Recently, terpenes have received extensive attention in the pharmaceutical field for their biological activities, including anti-inflammatory and antiapoptotic, and their inhibition of tumor proliferation and metastasis [4,5]. Currently, chemical synthesis and plant extraction are the main methods for terpene acquisition. However, the plant growth process is long, the product extraction process from plants is complex, and chemical synthesis is costly and environmentally unfriendly [6]. With the rapid advancements in synthetic biology and in-depth analysis of natural product biosynthesis pathways, the design of microbial cell factories for high value-added terpene synthesis [7,8] based on 2 natural terpene synthesis pathways, the mevalonate (MVA) pathway and the methyl-d-erythritol-4-phosphate (MEP) pathway [9,10], has become an emerging methodology. Terpene synthesis occurs mainly via the MVA pathway in eukaryotes and via the MEP pathway in prokaryotes [11] (Fig. 1). Recently, the oleaginous yeast *Yarrowia lipolytica* has been intensely employed for terpenoid production. This review aims to present both synthetic biology tools and established and nascent strategies for improving terpenoid production in *Y. lipolytica*.

**Fig. 1.**
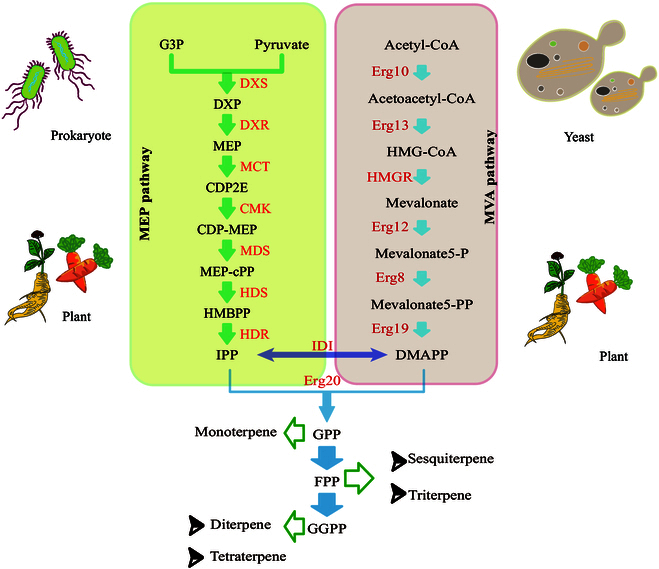
The terpene synthesis pathway. Terpenes are synthesized via the MEP pathway in prokaryotes and via the MVA pathway in eukaryotes such as yeast. Both of these synthesis pathways occur in plants. HDR, 1-hydroxy-2-methyl-2-(E)-butenyl-4-diphosphate reductase; HDS, 1-hydroxy-2-methyl-2-(E)-butenyl-4-diphosphate synthase; MDS, 2C-methyl-d-erythritol-2,4-cyclodiphosphate synthase; CMK, 4-diphosphocytidyl-2C-methyl-d-erythritol kinase; MCT, 4-diphosphocytidyl-2C-methyl-d-erythritol cytidylyltransferase; DXR, 1-deoxy-d-xylulose 5-phosphate reductoisomerase; DXS, 1-deoxy-d-xylulose 5-phosphate synthase; Erg10, acetyl-CoA C-acetyltransferase; Erg13, hydroxymethylglutaryl-CoA synthase; HMGR, hydroxymethylglutaryl-CoA reductase; Erg12, mevalonate kinase; Erg8, phosphomevalonate kinase; Erg19, mevalonate pyrophosphate decarboxylase; IDI, isopentenyl-diphosphate isomerase; Erg20, farnesyl diphosphate synthase.

## *Y. lipolytica* Is a Suitable Host for Terpene Synthesis

**Fig. 2.**
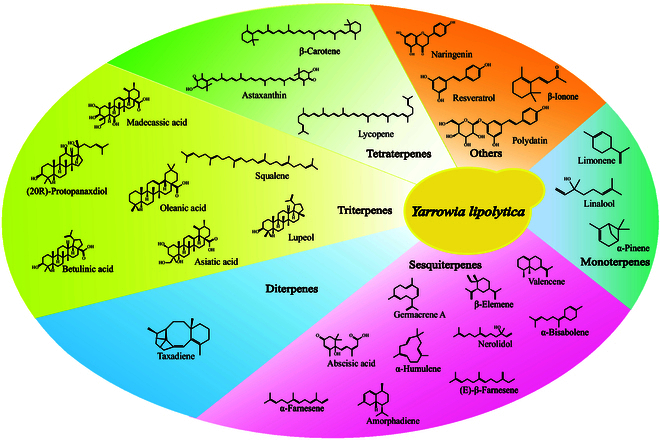
Synthesis of terpenes in *Y. lipolytica.*

Currently, the biosynthesis of functional terpenes and their derivatives has been successfully realized in microorganisms such as *Escherichia coli*, *Saccharomyces cerevisiae*, and *Y. lipolytica* [12,13]. The prokaryotic microorganism *E. coli* is the microorganism most commonly used in microbial cell factory construction for high value-added chemical production, but the lack of an endomembranal system may restrict the expression of membrane-anchored proteins such as cytochrome P450 enzymes (CYP450s) [14]. In comparison, yeast and other eukaryotic microorganisms are excellent for complex protein synthesis and bioactive substance production. Moreover, the development of rapid pathway assembly tools and dynamic regulation systems for metabolic networks has enabled the heterologous synthesis of natural compounds in yeast [15]. As an oleaginous yeast, *Y. lipolytica* has various advantages, such as salt tolerance, low-temperature resistance, and the ability to grow in low-pH environments. Metabolically, it can utilize a variety of substrates for growth such as glucose, waste cooking oil, and glycerol [16,17]. In addition, the high-flux tricarboxylic acid cycle (TCA) provides abundant acetyl-coenzyme A (CoA) for terpene synthesis, while its abundant lipid droplets and subcellular structure also provide storage sites for hydrophobic products [18]. Furthermore, *Y. lipolytica* has marked flux of the pentose phosphate pathway (PPP), which can provide NADPH (reduced form of nicotinamide adenine dinucleotide phosphate) for product synthesis [19]. Moreover, as a well-established chassis strain, there are clear genetic backgrounds and efficient gene editing tools for *Y. lipolytica* [16,17,19]. All these advantages of *Y. lipolytica* make it an excellent host. To date, a series of terpenes such as limonene [20–26], β-farnesene [25,27–30], squalene [25,31–33], and β-carotene [34–47] have been synthesized in *Y. lipolytica* [48] (Table 1) (Fig. 2). Among them, the yields of trans-nerolidol [49], α-farnesene [50], β-farnesene [29], and β-elemene [51] have the highest reported yields.

**Table 1.** Synthesis of terpenes and other products in *Y. lipolytica*

Compound	Strain	Carbon source	Strategies for terpenoid synthesis			Titer	Fermentation condition	Reference
			Up-regulated or overexpressed genes	Down-regulated genes	Knockout gene			
**Monoterpenes**								
Limonene	Po1f	Glucose, pyruvate	*TArLS*, *tSlNDPS1*, *HMG*, *ERG12*			23.6 mg/l	Shake flask	[22]
Limonene	Po1g	Waste cooking oil	*HMG*, *ClLS*, or *MsLS*			d-Limonene: 11.7 mg/l l-Limonene: 11.1 mg/l	5-l bioreactor	[23]
Limonene	Po1f	Citric acid, glycerol	*TArLS*, *tSlNDPS1*, *HMG*, *ERG12*			165.3 mg/l	bioreactor	[24]
Limonene	ATCC 20460	Glucose	*HMG*, *ERG12*, *ACL1*, *SeACS*, *IDI, ERG20^F88W-N119W^*, *PfLS*	*SQS*		35.9 mg/l	Glass tube	[25]
Limonene	Po1f	Lignocellulose hydrolysis product, citric acid	*ssXR*, *ssXDH*, *XKS*, *TArLS*, *tSlNDPS1*, *HMG*, *ERG12*			20.57 mg/l	Shake flask	[20]
Limonene	Po1f	Waste cooking oil	*ClLS* or *MsLS*, *HMG*, *IDI*, *tSlNDPS1*			d-Limonene: 91.24 mg/l l-Limonene: 83.06 mg/l	Shake flask	[26]
Linalool	Po1f	Citric acid, pyruvic acid	*AaLIS*, *HMG*, *IDI*, *ERG20^F88W-N119W^*			6.96 mg/l	Shake flask	[79]
α-Pinene	Po1f	Waste cooking oil, soybean oil, and lignocellulosic hydrolysate	*HMG*, *tSlNDPS1*, *tPtPS*, *ERG8*, *ERG12*, *MBP-ERG12*, *AMPD*			33.8 mg/l; 36.6 mg/l; 36.1 mg/l	Shake flask	[99]
**Sesquiterpenes**								
α-Farnesene	Po1h	Glucose, fructose	*SctHMG*, *IDI*, *MdFS-L-ERG20*			259.98 mg/l	1.5-l bioreactor	[81]
α-Farnesene	Po1f	Glucose	*BpHMG*, *ERG13*, *ERG12*, *IDI*, *ERG8*, *ERG19*, *GPPS*, *MdFS-L-ERG20*			25.55 g/l	1-l bioreactor	[50]
α-Farnesene	Po1f	Glucose, glycerol	*FS-L-ERG20*, *IDI*, *SctHMG1*, *HMG*, *ERG19*			2.57 g/l	5-l bioreactor	[100]
β-Farnesene	ATCC 20460	Glucose	*AaBFS*, *HMG*, *ERG12*, *ACL1*, *SeACS*, *IDI*, *ERG20*			955 mg/l	Glass tube	[25]
β-Farnesene	Po1f	Glucose	*AaBFS*, *tHMGR*, *ERG8*, *ERG10*, *ERG12*, *ERG13*, *ERG19*, *ERG20*, *IDI*		*DGA1-2*, *GUT2*, *POX3-6*,	22.8 g/l	2-l bioreactor	[28]
β-Farnesene	Po1f	Oleic acid, waste cooking oil	*ERG20*, *Aan^FSK197T-F180H^*, *ERG8*, *ERG12*, *ERG19*, *IDI*, *GPPS*		*DGA1-2*	35.2 g/l; 31.9 g/l	5-l bioreactor	[29]
β-Farnesene	Po1f	Lignocellulose hydrolysate	*AaBFS*, *tHMGR*, *spHMGR*, *ERG8*, *ERG19*, *ERG10*, *ERG13*, *ERG12*, *IDI*, *ERG20*			7.38 g/l	2-l bioreactor	[30]
β-Farnesene	Po1f	Glucose	*AaBFS*, *tHMGR*, *spHMGR*, *ERG8*, *ERG19*, *ERG10*, *ERG13*, *ERG12*, *IDI*, *ERG20*, *McMAE*, *MmACL*, *AMPD*, *YHM2*		*PFK*	28.9 g/l	2-l bioreactor	[27]
α-Humulene	Po1f	Glucose	*POT1*, *AcACHS2-PTS, RpHMG-PTS*, *ANT1*, *ERG12-PTS*, *ERG8-PTS*, *ERG20-PTS*, *ERG10-PTS*, *ERG13-PTS*, *ERG19-PTS*, *IDI-PTS*			3.2 g/l	5-l bioreactor	[88]
α-Humulene	Po1f	Waste cooking oil	*YALI0E32835*, *POT1*, *AcACHS2-PTS*, *RpHMG-PTS*, *ANT1*, *ERG12-PTS*, *ERG8-PTS*, *ERG20-PTS*, *ERG10-PTS*, *ERG13-PTS*, *ERG19-PTS*, *IDI-PTS*	*YALI0B21780*, *YALI0B21142*		5.9 g/l	5-l bioreactor	[101]
α-Humulene	Po1f	Glucose	*AcACHS2*, *tHMG1*, *AAD*, *ERG12*, *ERG8*, *ERG20*, *ERG10*, *ERG13*, *ERG19*, *IDI*	*ERG9*		21.7 g/l	5-l bioreactor	[75]
α-Santalene	ATCC 201249	Glucose	*ClSTS*, *ERG8*, *tHMG*			27.92 mg/l	5-l bioreactor	[102]
Abscisic acid	ATCC 20460	Glucose	*HMG*, *ERG12*, *ACL1*, *SeACS*, *IDI*, *ERG20*, *BcABA1-4*, *BcCPR1*, *POS5*, *AtDTX50*	*SQS*		263.5 mg/l	Deep well plate	[5]
Valencene	ATCC 20460	Glucose	*HMG1*, *ERG12*, *ACL1*, *SeACS*, *IDI*, *ERG20*, CnVS	*SQS*		113.9 mg/l	Glass tube	[25]
Amorphadiene	Po1g	Glucose	*AaADS*, *HMG*, *ERG12*, *POT1*, *PAT1*			171.5 mg/l	Shake flask	[103]
Amorphadiene	Po1g Po1f	Glucose	*POT1*, *PAT1*, *MFE2*, *AaADS*, *ACS2*, *tHMG1*, *SQS1-ADS*		*PAH1*, *DGA1-2*	71.74 mg/l	Shake flask	[91]
α,β,γ-Bisabolene	Po1g	Waste cooking oil	*HMG*, *GcABC-G1*, *Agα-BS*, *Zoβ-BS*, *Haγ-BS*			α-Bisabolene: 973.1 mg/l; β-Bisabolene: 68.2 mg/l; γ-Bisabolene:20.2 mg/l	Shake flask	[80]
α-Bisabolol	Po1f	Glucose	*POT1*, *MrBBS*, *tHMG*, *ERG20*	*SQS*		364.23 g/l	Shake flask	[104]
α-Bisabolene	Po1g	Waste cooking oil	*Agα-BS*, *MLS-HMGR*, *MPC2*, *PDA1*, *MGM1*, *GcABC-G1*			1058.1 mg/l	5-l bioreactor	[92]
α-Bisabolene	JMY1212	Glucose	*BiS*, *tHMG1*, *DGA2*, *GPD1*		*POX1-6*, *TGL4*	1243 mg/l	Shake flask	[105]
α-Bisabolene	Po1g	Waste cooking oil	*Agα-BS*, *HMG1*, *DGA1*, *OLE1*, *GcABC-G1*			1954.3 mg/l	Shake flask	[78]
Patchoulol	Po1f	Glucose	*PS1*, *tHMG1*, *IDI*, *ERG8*, *ERG10*, *ERG12*, *ERG13*, *ERG19*, *ERG20*, *ERG20-PS1*	*ERG9*		2.864 g/l	5-l bioreactor	[76]
Trans-nerolidol	Po1f	Glucose, glycerol	*HMG1*, *IDI*, *ERG8*, *ERG10*, *ERG12*, *ERG13*, *ERG19*, *ERG20*, *FaNES1-ERG20*			11.1 g/l	5-l bioreactor	[49]
Germacrene A	Po1f	Glucose	*dlGAS*, *dlGAS-ERG20*, *HMG1*, *IDI*, *ERG13*, *ERG12*, *ERG8*, *ERG19*, *FAA1*	*ERG9*, *ACC1*		39 g/l	5-l bioreactor	[74]
β-Elemene	Po1f	Glucose	*LTC2*, *tHMG1*, *IDI*, *ERG20*,	*ERG9*		5.08 g/l	5-l bioreactor	[51]
**Diterpenes**								
Taxadiene	Po1f	Glucose	*TASY*, *tHMG1*, *GGSP1*, *ERG20-GGPPS1*, *SUMO-TASY*	*ERG9*		101.4 mg/l	5-l bioreactor	[83]
**Triterpenes**								
Betulinic acid	ATCC 201249	Glycerol	*tHMG1*, *SQS*, *AtLUP1*, *MtCYP716A12-tAtATR1*			26.53 mg/l	Shake flask	[106]
Betulinic acid	ATCC 201249	Glucose	*RcLUS*, *BPLO*, *LjCPR*, *SQS*, *SQE*, *HMG*, *MFE1*			204.89 mg/l	Total triterpenes in shake flask	[107]
Lupeol	ATCC 201249	Glucose, pyruvate	*RcLUS*, *HMG*, *SQS*, *SQE*, *OLE1*		*PAH1*, *DGK1*	411.72 mg/l	Shake flask	[108]
Oleanic acid	ATCC 201249	Glucose	*tHMG*, *ERG20*, *SQS*, *GgBAS*, *MtCYP716A12-L-tAtATR1*			540.7 mg/l	5-l bioreactor	[109]
2,3-Oxidosqualene	ATCC 20460	Glucose	*HMG*, *ERG12*, *ACL1*, *SeACS*, *IDI*, *ERG20*, *SQS*, *SQE*	*ERG7*		22 mg/l	Deep well plate	[25]
Protopanaxadiol	ATCC 201249	Xylose	*SsXR*, *SsXDH*, *XKS*, *PgDS*, *PgPPDS-L-AtATR1*, *tHMG*, *ERG20*, *SQS*, *TKL*, *TAL*, *TX*		*POX1-3*	300.63 mg/l	5-l bioreactor	[110]
Squalene	Po1f	Glucose, citric acid	*HMG*, *ACL1*, *SeACSL641p*			10 mg/g DCW	Shake flask	[31]
Squalene	ATCC 20460	Glucose	*HMG, ERG12, ACL1, SeACS, IDI, ERG20*, *SQS*	*ERG7*		402.4 mg/l	Deep well plate	[22]
Squalene	Po1g	Glucose	*SQS*, *HMG*, *MnDH2*, *ACL2*			502.7 mg/l	Shake flask	[32]
Squalene	Po1f	Glucose	*HMG*, *DGA1*		*PEX10*, *URE2*	240.5 mg/l	Shake flask	[33]
Asiatic acid, madecassic acid, arjunolic acid	ST6512	Glucose	*CaCYP716C11p*, *CaCYP714E19p*, *CaCYP716E41p*			Madecassic acid: 0.1 mg/g DCW; Arjunolic acid: 9.1 mg/g DCW	Deep well plate	[111]
**Tetraterpenes**								
Astaxanthin	ST7403	Glucose	*XdcrtYB*, *XdcrtI*, *HMG*, *XdcrtE*, *PscrtW*, *PacrtZ*	*SQS*		54.6 mg/l	Deep well plate	[77]
Astaxanthin	ST7403	Glucose	*XdcrtYB*, *XdcrtI*, *HMG*, *XdcrtE*, *SsGGPPS*, *HpBKT*, *HpCrtZ*	*SQS*		285 mg/l	1-l bioreactor	[112]
Astaxanthin	ST7403	Safflower oil	*XdcrtYB*, *XdcrtI*, *HMG*, *XdcrtE*, *PscrtW*, *PacrtZ*	*SQS*		167 mg/l	1.5-l bioreactor	[82]
Astaxanthin	Po1f	Glucose	*PsCrtW-HpCrtZ-SKL*, *PsCrtW-HpCrtZ-OLEOSIN*, *PsCrtW-HpCrtZ-KDEL*, *SaGGPPS*, *McCarRP*, *McCarB*			858 mg/l	Shake flask	[113]
Canthaxanthin	Po1f	Glucose	*BsCrtW*, *McCarB*, *McCarRP*, *GGPPS*			36.1 mg/l	Shake flask	[114]
β-Carotene	Po1f	Glucose	*McCarB*, *McCarRP*, *ERG8*, *ERG10*, *ERG12*, *ERG13*, *ERG19*, *ERG20*, *GGPPS*, *tHMG*, *IDI*		*POX3-6*	4 g/l	2-l bioreactor	[34]
β-Carotene	ATCC 20460	Glucose	*McCarB*, *McCarRP*, *HMG*, *GGPPS*, *DGA2*, *GPD1*		*POX1-6*, *TGL4*	6.5 g/l	5-l bioreactor	[35]
β-Carotene	Po1g	Glucose	*EcAtoB*, *ScERG13*, *HMG*, *ERG8*, *ERG12*, *ERG19*, *ERG20*, *IDI*, *GGPPS*, *McCarB*, *McCarRP*			12 mg/g DCW	Shake flask	[36]
β-Carotene	S11073	Glucose	*McCarB*, *McCarRP*			75 mg/l	Shake flask	[37]
β-Carotene	ATCC 20460	Glucose	*HMG*, *ERG12*, *ACL1*, *SeACS*, *IDI*, *GGPPS*, *XdcrtYB*, *XdcrtI*	*SQS*		164 mg/l	Deep well plate	[25]
β-Carotene	Po1f	Glucose	*McCarB*, *McCarRP*, *GGPPS*, *HMG*, *ERG13*		*POX2-3*, *MFE*	4.5 g/l	5-l bioreactor	[38]
β-Carotene	Po1f	Glucose	*tHMG*, *BtCarB*, *BtCarRA*, *GGPPS*, *Hxk*, *ERG13*		*GUT2*	2.4 g/l	50-l bioreactor	[39]
β-Carotene	Po1f	Glucose	*tHMG*, *CarB*, *CarRP*, *GGPPS*, *DID2*		*GUT2*	2.01 g/l	5-l bioreactor	[40]
β-Carotene	IMUFRJ 50682	Glucose	*McCarB*, *McCarRP*, *GGPPS*			50.1 mg/l	Shake flask	[41]
β-Carotene	Po1f	Glucose	*tHMG*, *BtCarB*, *BtCarRA*, *GGPPS*, *DID2*		*GUT2*	2.6 g/l	5-l bioreactor	[42]
β-Carotene	Po1f	Glucose	*tHMG*, *GGPPS*, *BtCarRA*, *BtCarB*			1.7 g/l	5-l bioreactor	[43]
β-Carotene	Po1f	Glucose	*McCarB*, *McCarRP*		*NDT80*	12.5 mg/g DCW	Shake flask	[44]
β-Carotene	Po1f	Glucose	*AfGGPS*, *IDI*, *ERG8*, *ERG10*, *ERG12*, *ERG19*, *ERG20*, *VHb*, *McCarRP*, *GGPPS*, *McCarB*, *ScERG13*, *HMG*		*CLA4*, *MHY1*	7.6 g/l	1-l bioreactor	[45]
β-Carotene	Po1f	Glucose	*XdcrtI*, *XdcrtE*, *XdcrtYB*, *ACC1*, *tHMGR*			2.7 g/l	5-l bioreactor	[46]
β-Carotene, lycopene	Po1f	Glucose	*ScCK*, *AtIPK*, *ERG12*, *tHMG*, *ERG20*, *IDI*, *XdcrtE*, *CarB*, *McCarRP^Y27R^*, *McCarRP^E78K^*			β-Carotene: 39.5 g/l; Lycopene: 17.6 g/l	3-l bioreactor	[47]
Lycopene	H222	Glucose	*PaCrtB*, *PaCrtI*, *GGPPS*, *HMG*		*POX1-6*, *GUT2*	16 mg/g DCW	Shake flask	[115]
Lycopene	Po1f	Glucose, fructose	*PaCrtE*, *PaCrtB*, *PaCrtI*,			242 mg/l	1.5-l bioreactor	[116]
Lycopene	Po1f	Glucose	*HMG*, *PaCrtE*, *PaCrtB*, *PaCrtI*, *ERG8*, *ERG19*			213 mg/l	1-l bioreactor	[93]
Lycopene	Po1f	Glucose	*PaCrtE*, *PaCrtB*, *PaCrtI*, *AMPD*			745 mg/l	5-l bioreactor	[117]
Lycopene	Po1f	Glucose, palmitic acid	*PvIDI*, *LpCrtE*, *LpCrtB*, *LpCrtI*, *AtIPK*, *ScCHK*, *ERG20*			4.2 g/l	3-l bioreactor	[84]
**Others**								
β-Ionone	Po1f	Glucose	*McCarB*, *McCarRP*, *OfCCD1*, *SsNphT7*, *HpIDI*, *ERG8*, *ERG10*, *ERG12*, *ERG13*, *ERG19*, *tHMG*, *GPS*, *ERG20-GGPPS*			380 mg/l	2-l bioreactor	[118]
β-Ionone	Po1f	Glucose	*McCarB*, *McCarRP*, *PhCCD1*, *GGPPS*, *tHMG*, *ERG8*, *ERG10*, *ERG12*, *ERG13*, *ERG19*, *ERG20*, *IDI*, *BbPK*, *BsPTA*			0.98 g/l	3-l bioreactor	[87]
β-Ionone	Po1f	Organic waste hydrolysates	*McCarB*, *McCarRP*, *GGPPS*, *tHMG*, *ERG8*, *ERG10*, *ERG12*, *ERG13*, *ERG19*, *ERG20*, *IDI*, *BbPK*, *BsPTA*, *PhCCD1*			4 g/l	3-l bioreactor	[119]
Resveratrol	Po1f	Glucose	*FjTAL*, *Pc4CL1*, *ARO4^K221L^*, *ARO7^G139S^*, *YlARO1*, *YlARO3^K225L^*, *Pc4CL1-VvSTS*, *AtPAL-AtC4H*, *YlCYB5, AtATR2*, *CaFPK*, *BsPTA*		*DGA1*	22.5 g/l	5-l bioreactor	[120]
Polydatin	Po1f	Glucose	*RgTAL*, *At4CL*, *VvST1*, AroG*, TyrA*, *BbPK BsPTA*, *R3GAT*		*MHY1*, *DGA1-2*, *BGL2*, *EXG1*	6.88 g/l	Shake flask	[121]
Naringenin	Po1f	Glucose	*G2PS1*, *HsPKS1*, *AhSTS*, *OsCUS*, *RpBAS*, *SeSam8*, *Nt4CL*, *ARO4*			898 ± 19 mg/l	3-l bioreactor	[122]
Naringenin	Po1f	Glucose, xylose	*AtCHI*, *AtCHS*, *At4CL*, *RgTAL*, *XT*, *XR*, *XDH*, *XKS*		*DGA1*	715.3 ± 12.8 mg/l	Shake flask	[95]

### Elements for genetic editing in *Y. lipolytica*

#### Promoters

Promoters are critical components that regulate gene expression at the transcriptional level. To maintain cell growth and improve the production of target terpenoids, fine-tuning the expression of critical genes via promoter engineering is feasible. Currently, several promoters have been characterized in *Y. lipolytica* [52,53]. A series of endogenous promoters related to lipid metabolism have been identified [52,53]. The ranking of expression strengths was as follows: P_TEF_ > P_GAP_ > P_ACL2_ > P_ICL_ > P_IDH2_ > P_FAS1_ > P_DGA1_ > P_FAS2_ > P_ZWF1_ > P_POX4_ > P_ACC_ > P_IDP2_. After screening 81 endogenous promoters involved mainly in carbon and nitrogen metabolism, Wang et al. [53] classified them into 15 strong promoters, 41 medium promoters, and 25 weak promoters. Hybrid promoters, which are constructed mostly by fusing the upstream activation sequence (UAS) with the core promoter sequence, are another choice for gene expression [54,55]. Blazeck et al. [54] constructed a series of promoters by fusing the core sequence of P_TEF_ with 8 to 16 UASs, the strength of which increased with the number of fused UASs. Trassaert et al. [55] characterized the promoter of *EYK1*, an erythrosine kinase gene, and reported that the expression level of P_EYK1_ was increased in the presence of erythritol and erythrulose. Motivated by the above results, UAS_EYK1_ was identified, and inducible hybrid promoters were designed based on these results. Georgiadis et al. [56] isolated 3 strong promoters, P_H3_, P_ACBP_, and P_TMAL_, among which P_H3_ presented the strongest expression. Two hybrid promoters, UAS1B8-H3 and UAS1B8-TMAL, were obtained by attaching the minimal promoter sequences of H3 (260) and TMAL (250), respectively, to the 3′ end of the UAS (UAS1B8). These 2 hybrid promoters exhibited greater transcriptional activity compared with that of UAS1B8-TEF1 (136). Xiong and Chen [57] isolated 6 Cu^2+^-inducible promoters and 5 repressor promoters from the genome of *Y. lipolytica.* The repressor promoters showed relatively high activity in the absence of Cu^2+^, whereas the activity was almost suppressed in the Cu^2+^-abundant culture.

#### Terminators

Terminators are essential regulatory elements in transcription that take part in the transcription process and affect the stability of mRNAs [58,59]. In microorganisms, less effort has been expended on terminators than on promoters. CYC1t, XPR2t, and LIP2t are commonly used endogenous terminators in *Y. lipolytica* [58,59]. In addition to endogenous terminators, artificial terminators may be another choice for cassette construction. Compared with natural terminators, artificial terminators may be shorter in sequence and more effective, and the use of artificial terminators can also reduce the risk of unexpected homologous recombination (HR) caused by natural terminator reutilization [59].

#### Selection markers

In addition to other microorganisms, there are 2 screening markers for *Y. lipolytica*: nutritional defects and antibiotic resistance genes.

The most commonly used screening markers in *Y. lipolytica* genome editing are nutritional deficiency markers such as LEU2 and URA3 [58]. The use of LEU2 screening markers may affect lipid synthesis [28]. Because the presence of 5-fluoroorotic acid (5-FOA) leads to mutational inactivation of URA3 in nature, it is used for URA3 recycling in the gene editing process [60]. This process alleviates the limitations imposed by the limited number of screening markers. In addition, Hamilton et al. [61] reported that strains in which the acetamidase gene (*AMD1*) was knocked out could utilize acetamide as the sole nitrogen source, and those strains could be selected on acetamide medium (positive selection) or fluoroacetamide medium (negative selection).

*Y. lipolytica* is naturally sensitive to some antibiotics such as hygromycin B and nourseothricin. On the basis of these findings, antibiotic resistance genes have been used as screening markers, bridging the limitations associated with the limited number of nutrient-deficient markers [49,62]. Fickers et al. [62] designed the Cre/loxP recombination system in *Y. lipolytica* based on 2 screening markers, URA3 and hygromycin B, which are effective for genome integration and expression and marker recovery.

### Distinct gene expression approaches have been developed for *Y. lipolytica*

#### Free plasmid-based gene expression in *Y. lipolytica*

Currently, no natural free plasmid has been distinguished in *Y. lipolytica*. With the intensive study of metabolic engineering, researchers have constructed artificial free plasmids for *Y. lipolytica* [63,64]. The autonomously replicating sequence (ARS), which contains an origin (ORI) and a centromere (CEN), is particularly critical for artificial free plasmid construction. There are 4 main ARSs in *Y. lipolytica*: ARS1, ARS2, ARS18, and ARS68 [53,64] (Table 2).

**Table 2.** ARSs/CENs of plasmids commonly used in *Y. lipolytica*

Plasmids	ARS/CEN	Reference
JMP113	ARS68	[62]
P-UAS1B8-TEF	ORI1001, CEN1	[54]
CRISPR-cas9	ORI1001, CEN1	[65]
P-YaliA1	ORI1001, CEN1-1	[52]
ylAC	ORI3018, CEN3-1	[63]
P-mtORI	mtORI	[56]

Even when plasmid expression vectors can be constructed, the copy number and stability of these vectors may be low [65], which is not conducive to stable expression of heterologous genes.

#### Genome integration-based gene expression in *Y. lipolytica*

Genome integration of gene cassettes is the general way to maintain stable gene expression during metabolic engineering. There are 2 different methods of DNA integration in nature: HR and nonhomologous end joining (NHEJ) [66,67]. NHEJ is the preferred method for repairing DNA double-strand breaks (DSBs) in *Y. lipolytica*, whereas HR is the preferred method in *S. cerevisiae* [66,67].

To achieve efficient HR in *Y. lipolytica*, the homologous arms typically need to be longer than 1 kb. In addition to increasing the homologous arm length, HR efficiency can also be efficiently enhanced by the knockout or overexpression of specific genes. As the genes *KU70* and *KU80* are responsible for NHEJ, the knockout of these 2 genes is a common strategy [67]. HR efficiency was significantly improved after the knockout of *KU70*. The HR efficiency increased to 43%, even with only 50-base pair (bp) homology arms in the *KU70* knockout strain [67].

There are several superfamilies in *Y. lipolytica* that are involved in HR. HR efficiency can be increased by overexpressing genes of the RAD52 superfamily (*RAD51*, *RAD52*, *RAD54*, *RAD55/57*, *RDH54*, and *RAD59*), exonuclease genes involved in DSB terminal cleavage (*MRE11-RD50-XRS2* complex, *EXO1*, and *SGS1*-*DNA2*), and *SAE2* [68]. HR efficiency of up to 95% was achieved after overexpressing *ScRAD52* from *S. cerevisiae,* which is 6.5-fold greater than that of the wild-type strain and 1.6-fold greater than that of the *ku70* knockout strain.

For rapid metabolic pathway integration, gene integration at multiple loci may be an excellent choice. Zhou et al. [69] developed an efficient genetic tool based on the Cre/loxP system, in which the selection markers can be recycled. Lv et al. [70] developed another tool that combines the 26S ribosomal DNA (rDNA) loci and the Cre/loxP system. Liu et al. [71] developed a set of integrated vectors by taking advantage of the diversity of long terminal repeat sequences (LTRs) and rDNA sequences. Recently, another gene integration strategy was developed based on Du’s work on machine learning [72]. In addition, the development and application of the clustered regularly interspaced short palindromic repeats (CRISPR)/CRISPR-associated 9 (Cas9) system in *Y. lipolytica* has enabled rapid gene editing [73]. CRISPR-based tools have irreplaceable advantages in gene expression and dynamic biosensor construction [73].

## Strategies Used in *Y. lipolytica* Engineering for Terpene Accumulation

**Fig. 3.**
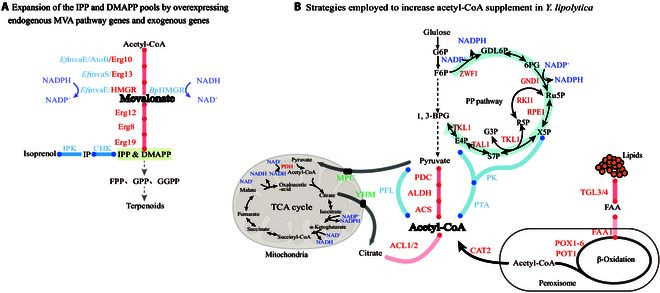
Terpenoid synthesis strategies for increasing the supply of precursors and cofactors in *Y. lipolytica.* (A) Expansion of IPP and DMAPP pools via overexpression of endogenous MVA pathway genes and exogenous genes. (B) Strategies employed to increase acetyl-CoA and cofactor supplementation in *Y. lipolytica*. The endogenous MVA pathway genes are in red, and the exogenous genes are in blue. TGL3/4, triacylglycerol lipase 3/4; FAA1, fatty acid-CoA synthetase 1; POX1-6, peroxisome acyl-CoA oxidase 1-6; POT1, 3-ketoacyl-CoA thiolase; PK, phosphoketolase; PTA, phosphotransacetylase; PDC, pyruvate decarboxylase; ALDH, aldehyde dehydrogenase; ACS, acetyl-CoA synthase; PFL, pyruvate-formate lyase; CAT2, peroxisomal carnitine acetyltransferase; IPK, isopentenyl phosphate kinase; CHK, choline kinase; MPC, mitochondrial pyruvate carrier; YHM, mitochondrial citrate carrier; ACL, ATP-citrate lyase; *Ef*mvaS, *Enterococcus faecalis* HMG-CoA synthase; *Ef*mvaE, *Enterococcus faecalis* acetoacetyl-CoA thiolase/HMG-CoA reductase; AtoB, *Escherichia coli* acetyl-CoA acetyltransferase; *Bp*HMGR, *Bordetella petrii* NADH-dependent HMGR; PDH, pyruvate dehydrogenase; ZWF1, glucose-6-phosphate dehydrogenase; PGLS, 6-phosphogluconolactonase; GND1, 6-phosphogluconate dehydrogenase 1; RPE1, d-ribulose-5-phosphate 3-epimerase 1; RKI1, ribose-5-phosphateketol-isomerase 1; TKL1, transketolase 1; TAL1, transaldolase 1.

Currently, the main strategies for metabolic engineering focus on improving metabolic flux toward target products, inhibiting competing pathways and balancing cofactors (Fig. 3).

### MVA pathway engineering for terpene production

#### Increased *HMGR* expression improved terpene production

Hydroxymethylglutaryl-CoA reductase (HMGR) is the rate-limiting enzyme of the MVA pathway, the overexpression of which has been shown to promote terpene accumulation [49,74]. HMGR is a membrane protein localized in the endoplasmic reticulum membrane and contains an N-terminal membrane anchoring sequence and a C-terminal catalytic domain [16]. Related studies have shown that the synthesis of terpenes can be effectively enhanced by the expression of the N-terminally truncated HMGR gene (*tHMGR*) [35,75]. Peng et al. [76] reported that patchoulol production was improved by increasing the copy number of *tHMGR*. However, after comparing the properties of *HMGR* and *tHMGR*, Kildegaard et al. [77] found that only the overexpression of *HMGR* was beneficial for β-carotene production. Similar conclusions were obtained in the syntheses of trans-nerolidol, α-bisabolene, and germacrene A [49,74,78]. These results indicate that the overexpression of full-length *HMGR* is effective for terpene synthesis, whereas the effect of *tHMGR* overexpression is uncertain. Furthermore, in most works demonstrating that the overexpression of *tHMGR* is effective, no attempt has been made to verify whether the overexpression of full-length *HMGR* works better.

#### Enhancing terpene synthesis by optimizing the expression of *ERG20*

Farnesyl diphosphate synthase (Erg20) is a key enzyme for farnesyl pyrophosphate (FPP) generation, and its overexpression facilitates terpene synthesis [74,76]. The fusion expression of *ERG20* and the sesquiterpene synthase (*STS*) gene has been shown to increase terpene synthesis. In Liu et al.’s work [74], the fusion expression of *dlGAS* and *ERG20* (*dlGAS-ERG20*) led to a 10.6% improvement in germacrene A production compared with that of the strain individually overexpressing *ERG20* and *dlGAS*. A similar conclusion was obtained in trans-nerolidol synthesis [49]. After comparing different linkers and constructing the 3-dimensional structure of the fusion protein, it was found that the distance between the active sites was minimized when *ERG20-PTS* was expressed, which is beneficial for the synthesis of patchoulol [76]. Although the fusion expression of *ERG20* and *STS* is an effective way to increase sesquiterpene productivity, the most suitable fusion order is different for diverse *STSs* [49,76], a result that may be related to spatial differences in the active sites of the *STSs*.

Naturally, Erg20 tends to generate FPP, which is not favorable for the synthesis of monoterpenes [79]. Related studies have shown that constructing mutants of *ERG20* is an effective strategy. In Cao et al.’s [79] work, linalool production was improved significantly by overexpressing *ERG20F88W-N119W* with *HMG1* and the isopentenyl-diphosphate isomerase (*IDI*) gene.

#### Improving terpene yield by overexpressing other MVA pathway genes

In addition to *HMGR* and *ERG20*, the overexpression of other genes in the MVA pathway is known to increase the metabolic flux of terpene synthesis. Zhao et al. [80] found that the individual overexpression of 5 MVA pathway genes increased production of α-bisabolene. It has also been shown that overexpression of *ERG13* is the most effective strategy for β-carotene production [39]. Lv et al. [40] found that the integration of 2 copies of *ERG13* in a strain improved β-carotenoid production by 259% to 8.41 mg/g dry cell weight (DCW). Isopentenyl-diphosphate isomerase (IDI) catalyzes the transformation of isopentenyl pyrophosphate (IPP) and dimethylallyl pyrophosphate (DMAPP), which play vital roles in the production of geranyl pyrophosphate (GPP) and FPP [16,79]. Yang et al. [81] reported that after the overexpression of *IDI*, the production of α-farnesene increased to 57.08 mg/l, which was 2.67-fold greater than that of the control strain. In addition to overexpressing certain rate-limiting genes in the MVA pathway, overexpressing all genes in the MVA pathway is also an effective strategy [51], which requires many gene integration sites.

#### Down-regulation of competitive pathways

There is substrate competition in the biosynthesis of squalene and other terpenes when FPP is used as a precursor [75,77]. To increase the metabolic flux of target products such as sesquiterpenes and β-carotenoids, decreasing the expression of the squalene synthase gene (*ERG9*) is a common strategy [51,82]. As squalene is a precursor for the synthesis of ergosterol, a cell membrane component that is essential for cell growth, the knockout of *ERG9* is unfeasible. Currently, weak promoter replacement and native promoter truncation are widely used strategies [51,74]. Xu et al. [83] used the relatively weak promoter P_ERG11_ to replace endogenous P_ERG9_ in the synthesis of taxadiene and increased the production of taxadiene from 0.27 to 0.31 mg/l. In addition, Liu et al. [74] truncated P_ERG9_ to 50 bp and produced 787.5 mg/l germacrene A, which was 49% greater than that of the control strain. Li et al. [51] used P_ERG1_ to replace endogenous P_ERG9_, and the β-elemene production of the modified strain was increased by 6% to 420 mg/l compared with that of the control strain. Guo et al. [75] replaced endogenous P_ERG9_ with the Cu^2+^ repressor promoter P_CTR1_ in α-humulene synthesis. Compared with the control strain, the modified strain produced 54.2% more α-humulene [75]. Similarly, Peng et al. [76] also successfully applied a Cu^2+^-inhibited promoter to increase patchoulol yield.

#### Introduction of a heterologous pathway or gene to increase terpenoid production

In addition to activating the endogenous MVA pathway, another way to increase terpene production involves the introduction of an exogenous pathway or enzymes. The isopentenol utilization pathway can effectively convert isoprenoid alcohols to IPP [84]. The contents of IPP and DMAPP increased 15.7-fold with the introduction of the isopentenol utilization pathway (AtIPK-ScCHK) [84]. The β-carotene concentration increased 27.2% after the overexpression of the exogenous genes *MvaE* and *MvaS* from *Enterococcus faecalis*, which are associated with MVA pathway synthesis [85]. MVA production increased to 1.96 g/l after overexpressing *AtoB* from *E. coli* and the NADH [reduced form of nicotinamide adenine dinucleotide (oxidized form)]-dependent HMGR gene (*BpHMGR*) from *Bordetella petrii* together with endogenous *ERG13* [50].

### Improving terpenoid production by increasing acetyl-CoA supplementation

Acetyl-CoA is an important intermediate metabolite in organisms and is the precursor for the MVA pathway. In addition to MVA pathway optimization, strengthening the acetyl-CoA supply is also an important strategy for terpenoid production [27,49]. Relevant studies have demonstrated that increasing the supply of acetyl-CoA in the cytoplasm contributes to terpene synthesis [27].

#### Enhancement of the endogenous pathway

A decrease in adenosine monophosphate (AMP) caused by the activation of adenosine monophosphate deaminase (AMPD) expression inhibits isocitrate dehydrogenase activity in the presence of excess carbon or in the absence of nitrogen, leading to citric acid accumulation [86]. Citric acid can be transported to the cytoplasm via the mitochondrial citrate carrier YHM2. Citric acid is further converted into acetyl-CoA via the adenosine triphosphate (ATP)-citrate lyase ACL [86]. Recently, Bi et al. [27] found that β-farnesene production reached 697 mg/l after overexpressing *AMPD*, *YHM2*, and *MmACL*, which was 16.7% greater than that of the control strain.

In addition, another strategy to increase cytoplasmic acetyl-CoA accumulation is reducing lipid synthesis and enhancing β-oxidation [29,74].

To reduce lipid synthesis, Liu et al. [74] reduced the activity of acetyl-CoA carboxylase (ACC) by constructing a mutant and overexpressing the fatty acid-CoA synthetase gene (*FAA1*) and the triacylglycerol (TAG) lipase gene (*TGL4*), which resulted in increased production of germacrene A. Moreover, the β-oxidation pathway was enhanced by the overexpression of the 3-ketoacyl-CoA thiolase gene (*POT1*), resulting in a 69% increase in β-farnesene yield compared with that of the control strain [29].

#### Introduction of heterologous acetyl-CoA synthesis pathways

In addition to the endogenous acetyl-CoA accumulation pathways, the heterologous cytoplasmic acetyl-CoA accumulation pathway is another option to facilitate terpene production [87]. The phosphoketolase (PK)–phosphotransacetylase (PTA) pathway, which involves phosphotransketolase (BbPK) from *Bifidobacterium bifidum* and phosphotransacetylase (BsPTA) from *Bacillus subtilis*, could be a reliable choice. Lu et al. [87] integrated the PK-PTA pathway into the genomes of strains overexpressing *tHMG1* and the geranylgeranyl diphosphate synthase gene (*GGS1*), generating strains that presented a 2.50-fold increase in β-ionone yield to 135.4 ± 3.8 mg/l. Bi et al. [27] achieved a 20% increase in the yield of β-farnesene to 810 mg/l using the same strategy. However, for the synthesis of α-humulene and germacrene A, this strategy appears to be ineffective [74,75]. In our former work, in a comparison of the 4 acetyl-CoA generation pathways, overexpression of the peroxisomal carnitine acetyltransferase gene (*CAT2*) was the most favorable strategy for increasing the trans-nerolidol titer [49].

### Balancing cofactor metabolism

Cofactors are important reduction factors that protect cells from oxidative stress and play important roles in enzymatic reactions and metabolic flux homeostasis, including the MVA pathway [88]. Because the MVA pathway requires large amounts of NADPH, modulating intracellular metabolism to produce more NADPH and integrating the NADH-dependent HMGR are the most commonly used strategies.

NADPH is generated in the PPP. By overexpressing glucose-6-phosphate dehydrogenase and 6-phosphogluconate dehydrogenase genes (*ZWF1* and *GND*), the yield of mycosporine-like amino acids can be effectively increased [89].

Malate synthase (MAE) can utilize both NAD^+^ and NADP^+^, the expression of which significantly affects cofactor homeostasis maintenance [27]. The malic enzyme of *Mucor circinelloides*, McMAE, is a NADP^+^-dependent malic enzyme [27]. Compared with that of the control strain, the engineered strain overexpressing *McMAE* produced 552 mg/l β-farnesene, a 27.5% improvement [27].

The overexpression of a NADH-dependent HMGR gene (*SpHMGR*) from *Silicibacter pomeroyi* can drive carbon flux into the MVA pathway while mitigating NADPH dependence [90]. Guo et al. [88] reported that the introduction of *SpHMGR* promoted the synthesis of α-humulene. Bi et al. [30] overexpressed the genes *tHMGR* and *SpHMGR* in *Y. lipolytica* simultaneously, which achieved the simultaneous utilization of NADH and NADPH for the synthesis of β-farnesene. β-Farnesene production was increased by 18.7% in the integrated engineered strain.

### Taking advantage of the subcellular structure

In addition to the above commonly used strategies, the advantages of subcellular structures have gradually attracted the interest of researchers.

Eukaryotes, including *Y. lipolytica*, contain a variety of organelles such as the endoplasmic reticulum, mitochondria, and peroxisomes. Metabolites such as acetyl-CoA are abundant in some organelles. Specific reactions are efficiently segregated from the cytoplasm, and as a result, competition for substrates from competing pathways is effectively reduced. Researchers have localized the synthesis pathway to specific organelles such as peroxisomes [23], the endoplasmic reticulum [91], and mitochondria [92], from which the target products can be synthesized efficiently.

#### Compartmentalizing metabolic pathways in peroxisomes

The peroxisome consists of a single membrane that allows the passage of low-molecular-weight compounds and is the location of β-oxidation [49,88]. It also serves to sequester toxic molecules from the cytoplasm and reduces substrate inhibition. Guo et al. [88] constructed an α-humulene synthesis pathway in peroxisomes by fusing pathway genes with the peroxisome-targeting signal (PTS). When the peroxisomal adenine nucleotide transporter (ANT1) was overexpressed, α-humulene production increased from 5.6 ± 0.3 mg/l to 565.0 ± 21.1 mg/l. In our former work, a peroxisomal compartment trans-nerolidol synthesis pathway was constructed in which the engineered strain generated 930 mg/l trans-nerolidol, which was 31% greater than that of the control strain [49]. These studies suggest that the compartmentalizing pathway in peroxisomes is an applicable strategy for terpenoid generation in *Y. lipolytica*.

#### Compartmentalizing metabolic pathways in mitochondria

Mitochondria have high fluxes of the TCA cycle, which can provide precursors for terpene synthesis. Compared with that of the control strain, 306-fold greater α-bisabolene production was achieved by integrating the synthesis pathway in mitochondria [92].

### Improving terpene accumulation via lipid biosynthesis engineering

Lipophilic compounds such as terpenes are stored intracellularly in liposomes [35,93]. Terpene production can be improved by increasing the lipid content. Lu et al. [78] effectively increased lipid accumulation by overexpressing *DGA1*, resulting in a yield of 844.6 mg/l α-bisabolene. Notably, more acetyl-CoA is consumed for lipid accumulation, which is also detrimental to terpene biosynthesis. Related studies have shown that knockout of the genes *DGA1* and *DGA2*, which are responsible for lipid synthesis, increased β-farnesene production [28,29].

While the above work provides exciting results in compartmentalization, morphology engineering, and cellular transport, these areas of research are still relatively unexplored in *Y. lipolytica*. It would be exciting for future studies to expand these promising areas.

## Challenges and Potential Solutions for Terpene Production in *Y. lipolytica*

The feasibility of terpene synthesis in *Y. lipolytica* has been demonstrated. Rapid advancements in gene editing technology and improvements in gene editing tools have promoted the application of *Y. lipolytica* in synthetic biology [16,17,19]. Although the construction and optimization of gene editing tools such as CRISPR/Cas9 have alleviated the limitations of low HR efficiency, there is still a large gap compared with model organisms such as *S. cerevisiae*. Thus, more efficient gene editing tools still need to be developed.

In addition, optimizing the protein folding efficiency of strains is a potential strategy for constructing terpenoid cell factories in the future. Many proteins are expressed during the construction of terpenoid cell factories, which increases the burden on the endoplasmic reticulum and thus affects protein folding efficiency. The unfolded protein response (UPR) in yeast cells is regulated by various transcription factors such as HAC1 [94]. Improving the efficiency of protein folding and conformational modification by regulating the expression of UPR-related transcription factors will contribute to the construction of microbial cell factories.

Optimizing the usage of carbon sources is also an emerging research direction in synthetic biology, which is important for the concrete implementation of sustainable development strategies. The use of nonfood-based carbon sources such as xylose [95] and methanol [96] has received increasing attention.

In addition, the dynamic regulation of product production by dynamic regulatory systems is another direction worthy of attention. The dynamic regulation system based on transcription factors or hormones has been applied to regulate the production of naringenin and other compounds [97]. Rapid screening of naringenin-producing strains was achieved by regulating leucine biosynthesis using the flavonoid transcriptional activator FdeR and its manipulator FdeO [98].

For terpene production, the combination of strategies mentioned above can effectively improve terpene yield, but the same strategy may produce very different results for diverse products. In the future, rationalizing metabolic network modeling at the genome scale could provide an in-depth perspective that may produce a series of universal strategies for terpene accumulation.
